# HIV-1 infection and circulating peripheral blood B cell subpopulations

**DOI:** 10.1186/1471-2334-14-S3-O6

**Published:** 2014-05-27

**Authors:** Ravinder Singh, Neema Negi, Bimal Kumar Das, Rakesh Lodha, SK Kabra, Madhu Vajpayee

**Affiliations:** 1 Department of Pediatrics, All India Institute of Medical Sciences, New Delhi, India; 2 Department of Microbiology, All India Institute of Medical Sciences, New Delhi, India

## Background

Progression of HIV-1 infection can be monitored by studying the frequency of B cell subpopulations which could serve as a better surrogate marker. The study evaluated the distribution as well as the frequency of B cell subsets in peripheral blood of HIV-1 infected Indian individuals.

## Methods

In HIV infected, ART naïve adults and healthy controls, frequency of B cell subpopulations were measured by flow cytometry. Difference between groups was compared using Student t test and *p* value of <0.05 was considered significant.

## Results

In HIV infected adults, a significant reduction in non-switched memory B cells (CD19+IgD+CD27+) was observed, compared to healthy controls (*p*=0.046). With ongoing viral replication and reduced CD4 count, an expansion in CD21^lo^CD27^-^ (tissue like memory) population was observed and the correlation was statistically significant (*p*=0.0004). The mean frequency of CD21^hi^CD27^hi^ (resting memory) was significantly higher in controls (*p*=0.0002) compared to HIV-1 infected adults, while tissue like memory were highly expanded in HIV infected adults compared to controls (*p*=0.0001). B cells in HIV-1 infected adults expressed higher frequency of CD95 compared to healthy controls (*p*=0.0004).

## Conclusions

HIV mediated alteration in B cell development and differentiation may result in loss of switched and non-switched memory B cells. Moreover, association of persistent viremia with expansion of CD21^lo^ tissue like memory cells suggests loss of CD21 expression as a marker of ongoing HIV replication. B cells in HIV-1 infected adults are more prone to *Fas* mediated apoptosis compared to healthy controls.

